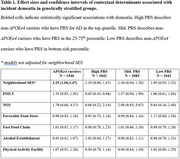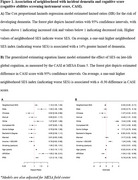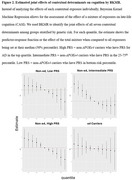# Integrating Contextual Determinants and Polygenic Risk to Examine Dementia and Cognition in the Multi‐Ethnic Study of Atherosclerosis

**DOI:** 10.1002/alz70860_101086

**Published:** 2025-12-23

**Authors:** Diane Xue, Elizabeth E Blue, Timothy M. Hughes, Wendy Post, Alison E Fohner

**Affiliations:** ^1^ University of Pennsylvania, Philadelphia, PA, USA; ^2^ University of Washington, Seattle, WA, USA; ^3^ Wake Forest University School of Medicine, Winston‐Salem, NC, USA; ^4^ Johns Hopkins Medicine, Baltimore, MD, USA

## Abstract

**Background:**

Structural determinants including neighborhood socioeconomic status (SES) and access to resources shape individual‐level risk factors like physical activity, education, and diet known to influence Alzheimer's disease (AD) and dementia risk. Despite the high heritability of AD, few studies have examined how neighborhood environment interacts with genome‐wide risk to influence dementia and cognition.

**Method:**

We assessed the associations of contextual determinants (SES, nitrogen dioxide, particulate matter < 2.5µg, and distances to the nearest favorable food stores, fast food chains, alcohol establishments, and physical activity facilities) on incident dementia and late‐life cognition (CASI) in the Multi‐Ethnic Study of Atherosclerosis. This diverse cohort included 5,713 participants, aged 45‐84 at baseline (2000‐2002; median follow‐up of 16.8 years). All models were adjusted for *APOE* genotype and polygenic risk score (PRS) for AD in addition to age, sex, study site, household income, education, smoking status, marital status, and race/ethnicity. The PRS (15 SNPs) was calculated using clumping and thresholding with r^2^ = 0.01 and a *p*‐value < 5E‐08. We compared the associations of contextual determinants stratified by genetic risk. We implemented Bayesian Kernel Machine Regression (BKMR) to explore the combined associations of all seven contextual exposures on late‐life cognition.

**Result:**

Lower neighborhood SES was associated with higher risk of incident dementia (HR_SES_ = 1.14; 95% CI: 1.05‐1.25, Figure 1) and lower late‐life cognition (β_SES_ = ‐0.30, 95% CI: ‐0.49 – ‐0.11, Figure 1) after adjusting for genetic risk and covariates. The association between neighborhood SES and dementia risk was strongest in the highest genetic risk group, though the interaction was not significant (Table 1). Other contextual determinants, including pollutants and built environment characteristics, were not associated with dementia or cognition after controlling for genetic risk. BKMR results revealed that the joint effects of social, built, and chemical environment on dementia were strongest in the intermediate genetic risk groups (Figure 2).

**Conclusion:**

Neighborhood SES was associated with incident dementia and late‐life cognition after adjusting for genetic risk factors and other individual‐level risks including education and household income, supporting the need for public health interventions targeting structural determinants of health to reduce dementia risk and improve cognitive outcomes.